# Machine learning techniques for diabetic macular edema (DME) classification on SD-OCT images

**DOI:** 10.1186/s12938-017-0352-9

**Published:** 2017-06-07

**Authors:** Khaled Alsaih, Guillaume Lemaitre, Mojdeh Rastgoo, Joan Massich, Désiré Sidibé, Fabrice Meriaudeau

**Affiliations:** 1 LE2I, CNRS, Arts et Métiers, Université Bourgogne Franche-Comté, 12 rue de la Fonderie, Le Creusot, France; 2 Centre for Intelligent Signal and Imaging Research (CISIR), Electrical & Electronic Engineering Department, Universiti Teknologi PETRONAS, 32610 Seri Iskandar, Malaysia

**Keywords:** DME detection, SD-OCT, Classification, HoG, LBP, BoW

## Abstract

**Background:**

Spectral domain optical coherence tomography (OCT) (SD-OCT) is most widely imaging equipment used in ophthalmology to detect diabetic macular edema (DME). Indeed, it offers an accurate visualization of the morphology of the retina as well as the retina layers.

**Methods:**

The dataset used in this study has been acquired by the Singapore Eye Research Institute (SERI), using CIRRUS TM (Carl Zeiss Meditec, Inc., Dublin, CA, USA) SD-OCT device. The dataset consists of 32 OCT volumes (16 DME and 16 normal cases). Each volume contains 128 B-scans with resolution of 1024 px × 512 px, resulting in more than 3800 images being processed. All SD-OCT volumes are read and assessed by trained graders and identified as normal or DME cases based on evaluation of retinal thickening, hard exudates, intraretinal cystoid space formation, and subretinal fluid. Within the DME sub-set, a large number of lesions has been selected to create a rather complete and diverse DME dataset. This paper presents an automatic classification framework for SD-OCT volumes in order to identify DME versus normal volumes. In this regard, a generic pipeline including pre-processing, feature detection, feature representation, and classification was investigated. More precisely, extraction of histogram of oriented gradients and local binary pattern (LBP) features within a multiresolution approach is used as well as principal component analysis (PCA) and bag of words (BoW) representations.

**Results and conclusion:**

Besides comparing individual and combined features, different representation approaches and different classifiers are evaluated. The best results are obtained for LBP$_{16 {\text{-}} \mathrm{ri}}$ vectors while represented and classified using PCA and a linear-support vector machine (SVM), leading to a sensitivity(SE) and specificity (SP) of 87.5 and 87.5%, respectively.

## Background

Eye diseases such as diabetic retinopathy (DR) and one of its complications, which is known as diabetic macular edema (DME), are the most common causes of irreversible vision loss in individuals with diabetes [1]. United States spent in health care and associated costs related to eye diseases almost $500 million  [1] while prevalent cases of DR were expecting to grow exponentially affecting over 300 million people worldwide by 2025. Early detection and treatment of DR and DME play a major role to prevent unfavorable effects such as blindness [2]. Screening programs on DR patients have been set up in many industrialized countries through the employment of fundus camera sometimes accompanied with optical coherence tomography (OCT) imaging. DME is characterized as an increase in retinal thickness within one disk diameter of the fovea centre with or without hard exudates and sometimes associated with cysts [2]. Spectral domain OCT (SD-OCT) scanner provides depth-resolved tissue structure information encoded in the magnitude and delay of the back-scattered light by spectral analysis [3]. It is an adequate tool compared to fundus photography for DME identification.

Automated diagnosis applied to OCT imaging is still at an early stage as only academic works have been published and no commercial products are yet available [4]. Most of the pioneer works on OCT image analysis have focused on the problem of retinal layers segmentation [5, 6] or specific lesion (e.g., cysts) segmentation as explained in [7, 8]. More recently, spectral domain OCT (SD-OCT) databases with their corresponding ground-truths were provided for benchmarking; for instance a challenge (OPTIMA) was organized as a satellite event of the MICCAI 2015 conference. The latest work of Fu et al. [9] shows promising results of quantitative grading of each individual slice of an OCT volume. The method relies on geometric and morphological features; however, the approach needs a standardization procedure which prevents it from being fully automated. It should be noted that the authors are providing the original images as a benchmark for the community. To the best of our knowledge, there are very few works like [9–11] addressing the specific problem of DME detection and its associated features detection from SD-OCT images. In this paper, we propose a solution for automated detection of DME on SD-OCT volumes.

## State-of-the-art on SD-OCT classification

This section discusses state-of-the-art methods for classification of SD-OCT volumes.

Srinivasan et al. proposed a classification method to distinguish normal, DME, and age-related macular degeneration (AMD) OCT volumes [10]. The SD-OCT volumes were enhanced by (1) reducing the speckle noise through a denoising method, which enforces the sparsity in a specific transform-domain and (2) flattening the retinal curvature. Then, edge information was extracted using histogram of oriented gradients (HoG) descriptor for each B-scan of a volume and later used to train a linear support vector machine (SVM). This method was evaluated on a dataset of 45 patients equally subdivided into three classes and resulted into a classification rate of 100, 100 and 86.7% for normal, DME and AMD patients, respectively. The dataset used by [10] is publicly available but it was already pre-processed (i.e., denoised, flattened, and cropped). Furthermore, this dataset does not offer a huge variability in terms of DME lesions. It also has different sizes for the OCT volumes, and some of them, without specifying which, have been excluded during the training; all these reasons prevent us from using this dataset to benchmark our work.

Venhuizen et al. [11] recently proposed a method to classify AMD and normal OCT volumes using bag of words (BoW) models. The features in their work were extracted from a set of keypoints detected from each individual B-scan. A 9 px × 9 px texton was extracted around each selected keypoint and its dimension was reduced, from 81 to 9 using principal component analysis (PCA). A dictionary or codebook was created by clustering the features extracted and each volume was represented in terms of a histogram represnting the codebook occurrences. These histograms were used as a final feature vector to train a random forest (RF) classifier; this classifier was evaluated on a dataset composed of 384 SD-OCT volumes leading to an area under the curve (AUC) of 0.984.

Liu et al. [12] proposed a methodology for detecting macular pathology in OCT images using local binary pattern (LBP) and gradient information as attributes. Each B-scan was aligned and flattened and a 3-level multi-scale spatial pyramid was created. Additionally, edges were detected using Canny detector on the same pyramid. Subsequently, an LBP histogram was extracted for each of the layer of the pyramid. All the obtained histograms were concatenated into a global descriptor whose dimensions were reduced using PCA. Finally, a SVM with an Radial Basis Function (RBF) kernel was used as classifier. The method achieved good results in detecting different pathologies such as DME or AMD, with an AUC of 0.93 using a dataset of 326 OCT scans.

Lemaître et al. proposed another method based on extracted LBP features from OCT images and dictionary learning using BoW models [13, 14]. BoW and dictionary learning were used to perform volume classification rather than B-scan. In this method, the OCT images were first pre-processed using (NLM) filtering to reduce the speckle noise. Then, the volumes were mapped into discrete sets of structures namely: local, when these structures correspond to patches; or global, when they correspond to volume slices or the whole volume. Texture features were extracted using different mapping techniques like LBP or three orthogonal planes (LBP-TOP) then represented per volume using histogram, PCA, or BoW. The final feature descriptors per volume are classified using RF classifier. Classifying DME versus normal volumes was applied on a balanced dataset of 32 SD-OCT volumes and the classification performance in terms of sensitivity (SE) and specificity (SP) of 87.50 and 75%, respectively was achieved.

On the same dataset, Sankar et al. [15] proposed a rather different approach, based on semi-supervised learning, to address the issue of an anomaly detection. In their method, the authors proposed a technique that not only allows the classification of the OCT volume, but also enables the identification of the abnormal B-scans inside the volume. This approach is based on modeling the appearance of normal OCT images with a Gaussian Mixture Models (GMM) and detecting abnormal OCT images as outliers. The classification of an OCT volume is based on the number of detected outliers. Testing on 32 OCT volumes, their method achieved SE and SP of 93 and 80%, respectively.

Albarrak et al. [16] proposed another classification framework to differentiate AMD and normal volumes. Each OCT slice undergoes two pre-processing routines: (1) a joint denoising and cropping step using the split Bregman isotropic total variation algorithm and (2) a flattening step by fitting a second-order polynomial using a least-squares approach. Then, LBP-TOP and HoG features are extracted and combined from individual sub-volumes. These features are concatenated into a single feature vector per OCT volume and its dimension was reduced using PCA. Finally, a Bayesian network classifier is used to classify the volumes. The classification performance of the framework in terms of SE and SP achieved 92.4 and 90.5%, respectively, This method’s results exceeded Liu et al. [12] results but using a dataset composed of 140 OCT volumes.

**Table 1:** Summary of the state-of-the-art methods for DME detection

References	Diseases			Data size	Pre-processing				Features	Representation	Classifier	Evaluation	Results
	AMD	DME	Normal		De-noise	Flatten	Aligning	Cropping					
Srinivansan et al. [10]	$\checkmark$	$\checkmark$	$\checkmark$	45	$\checkmark$	$\checkmark$		$\checkmark$	HoG		Linear-SVM	ACC	86.7%,100%,100%
Venhuizen et al. [11]	$\checkmark$		$\checkmark$	384					Texton	BoW, PCA	RF	AUC	0.984
Liu et al. [12]	$\checkmark$	$\checkmark$	$\checkmark$	326		$\checkmark$	$\checkmark$		Edge, LBP	PCA	SVM-RBF	AUC	0.93
Lemaître et al. [13]		$\checkmark$	$\checkmark$	32	$\checkmark$				LBP, LBP-TOP	PCA, BoW, Histogram	RF	SE, SP	87.5%, 75%
Sankar et al. [15]		$\checkmark$	$\checkmark$	32	$\checkmark$	$\checkmark$		$\checkmark$	Pixel-intensities	PCA	Mahalanobis-distance to GMM	SE, SP	80%, 93%
Albarrak et al. [16]	$\checkmark$		$\checkmark$	140	$\checkmark$	$\checkmark$			LBP-TOP, HoG	PCA	Bayesian network	SE, SP	92.4%, 90.5%

**Table 2:** Summary of the classification performance in terms of SE and SP in (%)

	Lemaitre et al. [13]	Sankar et al. [15]	Srinivasan et al. [10]	Liu et al. [12]	Venhuizen et al. [11]
SE	87.5	81.3	68.8	68.8	61.5
SP	75.0	62.5	93.8	93.8	58.8

Table 1 summarizes the relevant informations for all methods and Table 2 shows their performances on a common dataset [17].

**Table 3:** DME lesions types in SERI dataset

Type of lesions	SERI volumes No.	Type of lesions	SERI volumes No.
Vitreomacular Traction	4	Fluid HE and cystoid spaces	1
Cystoid spaces with hard exudates (HE) causing central retinal thickening	1	Cystoid spaces causing parafoveal retinal thickening	1
Cystoid spaces causing central and parafoveal retinal thickening	1	CSR with HE causing retinal thickening	2
CSR (subretinal fluid) causing central and parafoveal thickening	1	Cystoid spaces causing retinal thickening	3
Retinal thickening	2		

## Material

### SD-OCT data

The dataset used in the proposed algorithm has obtained an ethical approval and acquired by the Singapore Eye Research Institute (SERI), using CIRRUS TM (Carl Zeiss Meditec, Inc., Dublin, CA) SD-OCT device [13]. The dataset consists of 32 OCT volumes (16 DME and 16 normal cases). Each volume contains 128 B-scans with resolution of 1024 px × 512 px. All SD-OCT volumes are read and assessed by trained graders and identified as normal or DME based on evaluation of retinal thickening, hard exudates, intraretinal cystoid space formation, and subretinal fluid Within the DME sub-set, a large number of lesions has been selected to create a rather complete and diverse DME dataset (see Table 3).

### Source code

The source code associated with the experiments presented thereafter is available in GitHub.^1^

## Methods

**Fig. 1:**
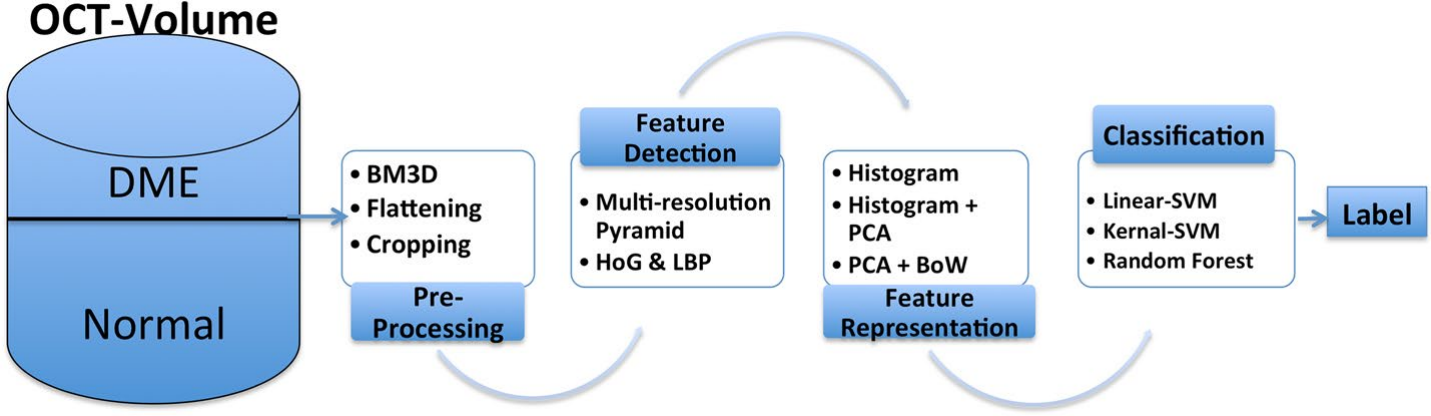
The pipeline is composed of: (1) pre-processing, (2) feature extraction, (3) feature representation, and (4) feature classification

Inspired by the previous methods, our classification pipeline is depicted in Fig. 1. This section explained into details each intermediate step.

### Pre-processing

**Fig. 2:**
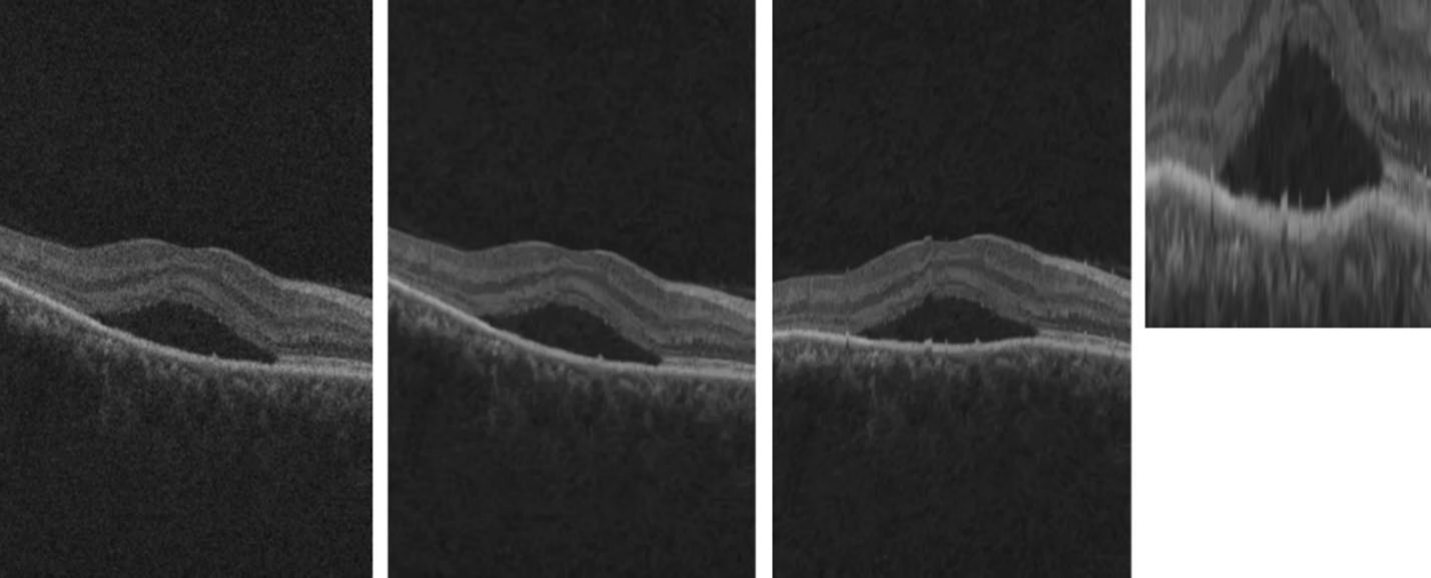
Example of SD-OCT preprocessed OCT images. (1) Original image, (2) OCT-denoised image, (3) OCT-flattened image, and (4) OCT-cropped image

Prior to feature extraction, the OCT volumes are pre-processed through denoising, flattening, and cropping as shown in Fig. 2.

In the first step, speckle noise is attenuated through an image denoising strategy. Different denoising methods have been implemented and tested on synthetic images as well as on SD-OCT B-scan. The latter type of images are further processed by the layer segmentation algorithm developed by Garvin et al. [6]. Among the tested methods, a set of conventional filters are investigated: (1) mean filter, (2) median filter, (3) local statistics filter (i.e. Lee filter [18]), (4) hard and soft thresholding in wavelet domain [19], (v) NLM [20], (BM3D) [21], k-SVD [22], a subspace technique [23], the (PGPD) [24] and the extension of NLM specifically designed for speckle noise, known as Optimized Bayesian NLM (OB-NLM) [25]. Each filter was optimized and the results were based on quantitative evaluations as well as qualitative evaluations (i.e., layers identification) while applying on SD-OCT images.

The images are then flattened and cropped similarly to [10] and the only difference relies on the fact that the second order polynomail fitting of the retinal pigment epithelium (RPE) was performed in conjunction with random sample consensus (RANSAC) algorithm. In the axial dimension, all images are cropped 325 px from over the RPE layer and 30px under the RPE. In the lateral dimension, all images are cropped 340 px to the center.

### Features detection

**Fig. 3:**
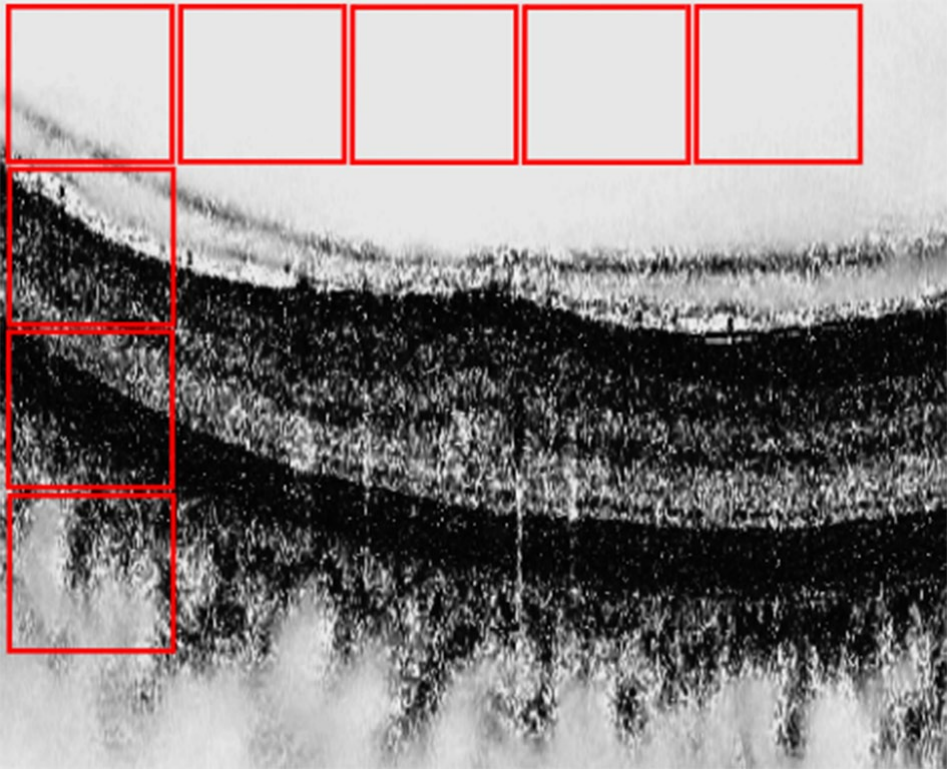
Local mapping. Example of non-overlapping windows on 2D slices

HoG features [26] and LBP features [27] were extracted from four levels using a multiresolution Gaussian image pyramid. LBP features were extracted from 32 px × 32 px non-overlapping patches (see Fig. 3). Additionally, rotation invariant and uniform (-ri) LBP features with various sampling points (i.e., $\{8,16,24\}$) with respect to different radius, (i.e., $\{1,2,3\}$), as well as non rotation invariant (-nri) LBP were extracted.

The number of patterns ($LBP_{\#\mathrm{pat}}$) for each configuration is reported in Table 4. Therefore, each slice is described by a feature vector which its size is equal to the number of patches multiply by the number of patterns ($LBP_{\#\mathrm{pat}}$) as reported on Table 5.

**Table 4:** Number of patterns (${\text{LBP}}_{\#\mathrm{pat}}$) for different sampling points and radius ({P, R}) of the LBP descriptor

Sampling point for a radius ({P, R})			
	{8, 1}	{16, 2}	{24, 3}
${\text{LBP}}_{\#pat}$	10	18	26

**Table 5:** Final LBP descriptor size per B-scan, after building the image pyramid for different sampling points and radius ({P, R}) of the LBP descriptor

Sampling point for a radius ({P, R})			
	{8, 1}	{16, 2}	{24, 3}
Feature vector size per B-Scan	180	324	468

HoG features were extracted with 4 px × 4 px cell size and 2 px × 2 px block size with $1\mathrm{px}$ overlap. Furthermore, the gradient orientation is discretized using 9 bin histogram resulting in feature vector size described in Table 6

**Table 6:** Final HoG descriptor size per B-scan, after building the image pyramid

Level of the pyramid				
	{1}	{2}	{3}	{4}
Feature vector size per B-Scan per level	266,112	63,468	15,120	3240
Total vector size per B-Scan	347,940			

### Feature representation

The LBP and HoG features from patches using the multiresolution image pyramid were first represented in terms of concatenated histograms (later called to “Histogram” for this configuration).

This method resulted in a high dimensional feature space; therefore PCA was used to reduce the number of dimensions of the concatenated histograms (later called “Histogram + PCA” for this configuration), resulting in a single feature vector per B-scan, subsequently a feature matrix per volume. Therefore, with the aim of providing a feature vector per volume, BoW approach was used in the last representation. Using the previously represented features (Histogram + PCA), BoW approach learned a dictionary and represented each volume by a histogram which captured the codebook occurrences (later called “Histogram + PCA + BoW” for this configuration).

### Classification

Three different classifiers were used for comparison: RF, linear-SVM, and kernel-SVM. A similar classification strategies as in [10] were used for the first two configurations, hence feature descriptors were used to train the classifiers in order to classify each B-scan. Subsequently, the SD-OCT volume classification was achieved based on the total number of diseased B-scans detected per volume, using the majority voting rule. Regarding the last representation using BoW, the volume classification was directly performed as the histogram of the visual words was built for each SD-OCT volume.

## Experiments and results

For the denoising, as shown in Table 7, BM3D led to the best peak signal-to-noise ratio (PSNR) and reduced noise on the SD-OCT image without affecting the key components (i.e., the layers on the SD-OCT images) compared to the other methods. These results are in adequation with those recently published by Fu et al. [9].

Once, the volumes were preprocssed using BM3D, the experiments were divided into two categories. *Exp1* tested different configurations leading to first B-scan and finally volume classification. Therefore, Histogram and Histogram + PCA representations of individual features as well as Histogram + PCA representation of the combined features were evaluated in this experiment. Later, only the feature representations leading to the best classification performance from *Exp1* were used in *Exp2*, in conjunction with BoW, to perform a direct volume classification.

As previously mentioned rotation invariant (-ri) and non-rotation invariant (-nri) LBP features with various radius, {8,16, 24}, were tested. However, it was observed that LBP-ri provided a better results and therefore only the results obtained with this configuration were mentioned in the following part.

Both experiments were validated using leave-two-patients-out method, such that at each cross-validation iteration, a DME and normal volume were kept out to test while the remaining volumes were used to train. Thus, a total of 16 cross-validations were performed. The results are reported in terms of SE and SP. Tables 8 and 9 show the results from *Exp1* for individual and combined features.

**Table 7:** PSNR (*dB*) for denoising algorithms considering speckle noise on synthetic images

Technique	Lena	Cameraman	Baboon
Mean	28.73	22.38	*28.84*
Median	27.82	22.11	27.82
Lee	27.47	28.08	20.97
Wavelet	28.36	28.49	20.97
Subspace	28.31	26.33	25.42
BM3D	*32.51*	*33.37*	24.12
k-SVD	31.29	30.83	25.90
PGPD	31.57	32.55	25.84
OB-NLM	30.10	30.94	25.03

The configurations which led to the best classification performance are highlighted in italic. These configurations were further tested in *Exp2* (see Table 10) using BoW representation. The optimal number of words has been selected heuristically while the number of components when applying PCA has been set to 40 and 20 for HoG and LBP descriptors, respectively, such that the most discriminative components are kept. PCA dimensions were selected empirically after a number of trials.

**Table 8:** *Exp1*—classification of individual features while represented using Histogram and Histogram + PCA

Classifier	Metric	Individual Features							
		Histogram				Histogram + PCA			
		HoG	${\text{LBP}}_{8{\text{-}}\mathrm{ri}}$	${\text{LBP}}_{16 {\text{-}} \mathrm{ri}}$	${\text{LBP}}_{24 {\text{-}} \mathrm{ri}}$	${\text{HoG}}^{~\mathrm{PCA}}$	${\text{LBP}}_{8 {\text{-}} \mathrm{ri}}^{~\mathrm{PCA}}$	${\text{LBP}}_{16 {\text{-}} \mathrm{ri}}^{~\mathrm{PCA}}$	${\text{LBP}}_{24 {\text{-}} \mathrm{ri}}^{~\mathrm{PCA}}$
Linear-SVM	SE	68.7	62.5	75.0	68.7	75.0	*87.5*	75.0	81.2
	SP	87.5	81.2	75.0	87.5	75.0	*87.5*	75.0	81.2
RBF-SVM	SE	93.7	93.7	87.5	87.5	12.5	81.2	*81.2*	75.0
	SP	6.2	25.0	25.0	50.0	87.5	81.2	*87.5*	87.5
RF	SE	62.5	75.0	*81.2*	68.7	56.2	75.0	*75.0*	75.0
	SP	100.0	81.2	*87.5*	93.7	93.7	81.2	*93.7*	93.7

**Table 9:** *Exp1*—classification of combined features using Histogram + PCA representation

Metric of combined features				
		HoG$^{~PCA}$ +		
Classifier	Metric	LBP$_{8{\text{-}}\mathrm{ri}}^{\mathrm{PCA}}$	HoG$_{16{\text{-}}\mathrm{ri}}^{\mathrm{PCA}}$	HoG$_{24{\text{-}}\mathrm{ri}}^{\mathrm{PCA}}$
Linear-SVM	SE	68.7	*75.0*	68.7
	SP	81.2	*87.5*	87.5
RBF-SVM	SE	68.7	18.7	0
	SP	81.2	93.7	100.0
RF	SE	62.5	*75.0*	62.5
	SP	81.2	*87.5*	87.5

## Discussion

Evaluations of individual features (see Table 8) show that the dimensionality reduction of the features and the use of Histogram + PCA representation improved the results of B-scan classification. The reason for that, we have only 30 (no. of volumes, two left out for testing) multiply by 128 (no. of B-scan per volume) points in the space to be classified, while the dimension space is huge as shown in Tables 5 and 6. Using only Histogram representation, RF classifier led to the best performance followed by linear-SVM. RBF-SVM classifier had the lowest performance for all the individual features due to overfitting. The performance was improved when the number of dimensions were reduced using PCA. Using the second representation the gap between the classifiers were reduced and the classification performances obtained were similar. Comparing individual features, LBP proved to be more discriminative than HoG features. This could be due to the rotation invariant property of LBP in comparison to non-invariant HoG descriptors.

Based on Table 9, the combination of LBP and HoG features did not improve the results but decreased the performance of individual features, the reason could be due to the higher dimensionality of the LBP^PCA^ + HoG^PCA^. In this test, RF and linear-SVM had similar performance while RBF-SVM was overfitting.

As shown in Table 2, various methods were tested using the same dataset. The pipeline applied by these methods vary in terms of each step, denoising, features extraction and classifying, which appear in disparate results (refer to state-of-the-art section)

**Table 10:** *Exp2*—classification results using Histogram + PCA + BoW representation

Histogram + PCA + BoW				
			Metric	
	Classifier	# Words	SE	SP
LBP$_{8{\text{-}}\mathrm{ri}}^{\mathrm{PCA}}$	Linear-SVM	10	62.5	75.0
LBP$_{16{\text{-}}\mathrm{ri}}^{\mathrm{PCA}}$	RBF-SVM	30	*81.2*	*50.0*
LBP$_{16{\text{-}}\mathrm{ri}}$	RF	40	56.2	50.0
LBP$_{16{\text{-}}\mathrm{ri}}^{\mathrm{PCA}}$	RF	50	68.7	50.0

To conclude with *Exp1*, the highest classification performance was achieved using: ${\text{LBP}}_{8{\text{-}}\mathrm{ri}}^{\mathrm{PCA}}$ and linear-SVM, ${\text{LBP}}_{16{\text{-}}\mathrm{ri}}^{\mathrm{PCA}}$ and RBF-SVM, ${\text{LBP}}_{16{\text{-}}\mathrm{ri}}$ and RF, and finally ${\text{LBP}}_{16{\text{-}}\mathrm{ri}}^{\mathrm{PCA}}$ and RF classifier. These configurations were later tested in *Exp2* using BoW representation. The results obtained from *Exp2* showed that Histogram + PCA + BoW representation led to lower the performance results. In fact, this approach represented each volume in terms of visual-B-scans rather than visual-patches or visual-sub-volumes, which could be a reason why BoW failed.

## Limitations of study

Although this study shows some promising results, some limitations have to be raised. The current study is a proof of concept based on a rather small dataset. Additional experiments need to be carried out on a larger set using the post experiment analysis, to show the robustness of our approach. The classification approach is the same as Srinivasan et al.  [10], training is done at the volume level and testing at the slide level with a majority vote to classify the volume. This implies that, DME volume (for training and testing) should contain more than half of the slides having presence of DME. We are currently working on Multiple instance learning to address this aspect  [7] and reinforce the training approach. Furthermore, the best classification performance with a sensitivity and a specificity of 87.5 and 87.5%, respectively, show that our method is still not ready for clinical purpose, with a too large false positive detection. It can be noted that the decision threshold of the classifier could be moved to increase the specificity of our approach to the detriment of the sensitivity. Despite our wish to foster Open Source and Data initiatives, the data used in this study are currently not available publicly to third-party, in the contrary to our implementation available in GitHub.

## Conclusion

We presented an automatic classification framework for SD-OCT volumes in order to identify DME versus normal volumes. In this regard, we investigated a generic pipeline including preprocessing, feature detection, feature representation, and classification. Besides comparing individual and combined features, different representation approaches and different classifiers were evaluated. The best results were obtained for ${\text{LBP}}_{16{\text{-}}\mathrm{ri}}$ vectors while represented and classified using PCA and linear-SVM. As future work, we would like to extend the dataset in order to make it more challenging as well as also making it public.

## Footnotes

1
https://github.com/I2Cvb/alsaih-2016-sep.
